# A placebo-controlled Phase 2 trial of E6011, anti-human fractalkine monoclonal antibody, in primary biliary cholangitis

**DOI:** 10.1016/j.jtauto.2025.100283

**Published:** 2025-03-20

**Authors:** Atsushi Tanaka, Masanori Abe, Tadashi Namisaki, Shinji Shimoda, Mikio Zeniya, Akio Ido, Hitoshi Yoshiji, Hiromasa Ohira, Kenichi Harada, Yuko Kakuda, Atsushi Umeda, Yuki Kamiya, Yukari Higashine, Seiichiro Hojo, Toshio Imai, Tetsu Kawano, Yasuni Nakanuma, Hirohito Tsubouchi

**Affiliations:** a Department of Medicine, Teikyo University School of Medicine, Tokyo, Japan; b Department of Gastroenterology and Metabology, Ehime University Graduate School of Medicine, Ehime, Japan; c Department of Internal Medicine, Nara Medical University, Nara, Japan; d Third Department of Internal Medicine, Kansai Medical University, Osaka, Japan; e Gastroenterology, Akasaka Sanno Medical Center, International University of Health and Welfare, Tokyo, Japan; f Digestive and Lifestyle Diseases, Kagoshima University Graduate School of Medical and Dental Sciences, Kagoshima, Japan; g Department of Gastroenterology, Fukushima Medical University School of Medicine, Fukushima, Japan; h Department of Human Pathology, Kanazawa University Graduate School of Medical Sciences, Ishikawa, Japan; i Division of Pathology, Shizuoka Cancer Center, Shizuoka, Japan; j EA Pharma Co., Ltd., Tokyo, Japan; k Eisai Co., Ltd., Tokyo, Japan; l KAN Research Institute, Hyogo, Japan; m Advanced Therapeutic Target Discovery, Kobe University Graduate School of Medicine, Hyogo, Japan; n Gastroenterology, Nichinan-City Chubu Hospital, Miyazaki, Japan; o Department of Pathology, Fukui Saiseikai Hospital, Fukui, Japan; p Department of Gastroenterology and Hepatology, Kagoshima City Hospital, Kagoshima, Japan

## Abstract

**Background:**

While ursodeoxycholic acid (UDCA) remains the first-line therapy for primary biliary cholangitis (PBC), the autoimmune nature of PBC underscores the need for treatments targeting immunological pathways that may achieve a cure. E6011, a novel humanized anti-fractalkine monoclonal antibody, has emerged as a potential therapeutic option for PBC. We conducted a randomized, placebo-controlled, double-blind study to evaluate the efficacy and safety of E6011 in patients with PBC with an incomplete response to UDCA.

**Methods:**

The study was composed of 12-week Double-Blind Phase (placebo, E6011 10 mg/kg/month, 15 mg/kg/month, or 10 mg/kg/every other week [eow]) followed by a 52-week Open-Label Phase. The primary endpoint was the percent change in alkaline phosphatase (ALP) at Week 12.

**Results:**

A total of 29 patients were enrolled. Histological evaluation at screening revealed that 83 % of the enrolled patients were classified as Stage 4 according to the Nakanuma Classification. The mean percent changes in ALP at Week 12 were +0.45 % in the placebo, +0.65 % in the 10 mg/kg/month, +1.23 % in the 15 mg/kg/month and +1.19 % in the 10 mg/kg/eow, with no observed trends toward ALP reduction in the E6011 treatment. Based on the interim analysis, the study was discontinued due to a lack of the efficacy. E6011 was generally safe and well tolerated.

**Conclusion:**

This study of E6011 failed to meet the primary endpoint in patients with PBC with an incomplete response to UDCA. The advanced histological severity present in more than 80 % of patients at baseline may have contributed to these findings.

## 1 Introduction

Primary biliary cholangitis (PBC) is a chronic, cholestatic, and slowly progressing disease that can eventually result in liver failure without appropriate treatment [1]. Although etiology of PBC has not been fully understood, robust evidence indicates that autoimmune reactions targeting intrahepatic biliary epithelial cells (BECs) play a central role in its etiopathogenesis. Indeed, PBC is considered to be a model autoimmune disease, because of the presence of disease-specific autoantibodies [antimitochondrial autoantibodies (AMAs)], intense infiltration of mononuclear cells in the vicinity of bile ducts, and a high prevalence of comorbid autoimmune diseases [2].

On the other hand, the main therapeutic targets of PBC are alleviation of cholestasis, not manipulation of aberrant autoimmunity against BECs, despite its autoimmune nature [3]. The first-line treatment of PBC is ursodeoxycholic acid (UDCA), consistently recommended by clinical practice guidelines [[4], [5], [6]], and indeed the long-term effectiveness of UDCA in patients with PBC was demonstrated by the real-world data [7]. For those with an incomplete response to UDCA, about 20–30 % of patients with PBC, obeticholic acid (OCA), a farnesoid X receptor (FXR) agonist, was officially approved in 2016, based on the result of successful phase 3 clinical trial [8]. Recently, peroxisome proliferator-activated receptor (PPAR) agonists have been attracting attention as an alternative second-line treatment. On the basis of currently available data, bezafibrate, pan (α-δ-γ)-PPAR agonist, stands out as the only PPAR agonist capable of combining a strong short-term biochemical response with a high rate of ALP normalization, improved pruritus and improved long-term outcomes [[9], [10], [11]]. Furthermore, two PPAR agonists, seladelpar (PPARδ agonist) and elafibranor (PPARα and δ agonist), emerged as novel and promising drugs in 2024, as the phase 3 trials of these two met biochemical endpoints with minimal adverse effects [12,13], and recently these two have been officially approved by the United States Food and Drug Administration. Nevertheless, these agents targeting cholestasis is not capable of facilitating a “cure” of PBC and need to be life-long administered to the patients. Since PBC is an autoimmune disease, it is an essential need to cure PBC by suppressing aberrant autoimmune reactions against BECs.

Fractalkine (FKN) is a membrane-bound chemokine, and the expression of FKN in the vascular endothelial cells is strongly induced by inflammatory stimulation. It binds to CX3CR1 expressed in monocytes, macrophages and killer lymphocytes to play important roles in intercellular cell adhesion and migration of these cells [14,15]. This FKN/CX3CR1 signal has been suggested to be closely related to the pathology of inflammatory disorders including inflammatory bowel disease and rheumatoid arthritis [[15], [16], [17], [18]]. Patients with PBC show increased expression of FKN in the biliary epithelial cells, as well as aggregation of monocytes and lymphocytes with CX3CR1 expression within the damaged bile duct, suggesting that the FKN/CX3CR1 signal also plays an important role in the pathogenesis and progression of PBC [19].

E6011 is the first humanized anti-FKN monoclonal antibody. In concanavalin A-induced hepatitis mouse models, which are used widely as autoimmune hepatic disorder models, a significant increase was observed for both the plasma alanine aminotransferase (ALT) and aspartate aminotransferase (AST) for the control group, whereas a significant suppressive effect was observed in the group administered 5H8 (administration through the tail vein), which is an anti-mouse FKN monoclonal antibody [20]. This result suggests that E6011 may suppress the FKN-induced migration of inflammatory cells and lymphocytes in the liver, and therefore could become a powerful treatment drug for PBC that is potentially able to bring about cure of the disease. A phase 1 clinical study in healthy subjects indicated that intravenous infusion of E6011 at single ascending dose from 0.0006 to 10 mg/kg was safe and well-tolerated. Moreover, Phase 1/2 open-label trials for patients with rheumatoid arthritis and Crohn's disease have already been conducted, Japanese Phase 2 study completed in patients with rheumatoid arthritis and global Phase 2 study completed in patients with Crohn's disease [[21], [22], [23], [24], [25]].

Based on the above background, we conducted a Phase 2 clinical study in Japan to evaluate the efficacy, safety and PK of E6011 in patients with PBC with an incomplete response to UDCA.

## 2 Patients and methods

### 2.1 Trial outline and ethics

This was a placebo-controlled, randomized, double-blind, multicenter, parallel-group comparison phase 2 study that assessed the efficacy and safety of E6011 in Japanese patients with PBC with an incomplete response to UDCA. The study was registered at http://clinicaltrials.gov (NCT03092765), and was conducted between March 2017 and August 2018 at 46 institutions in Japan. The protocol was reviewed and approved by the individual Institutional Review Boards of each institution. Written informed consents were obtained from all subjects prior to enrolment. This study was conducted in compliance with the Good Clinical Practice guidelines and ethical principles based on the Declaration of Helsinki.

### 2.2 Patients

Subjects were male or female out-patients, aged ≥20 and < 80 years old, with PBC who met at least two of the followings: a) elevation of cholestatic enzymes, b) positivity for AMA, c) histology compatible with PBC. Patients were required to have a constant dose administration of UDCA (600 mg/day or higher; 600 mg/day corresponds to the Japanese normal dose) for at least 6 months before the start of the screening but remained ALP level 1.67 to 10 times higher than the upper limit of normal (ULN) at screening and Week 0. Key exclusion criteria were as follows; patients with elevated level of a total bilirubin higher than 2 times the ULN, an AST or ALT higher than 5 times the ULN, creatinine higher than 1.5 mg/dL at screening; patients with a history or presence of decompensated cirrhosis events including variceal bleeding, encephalopathy at a coma level of II or higher, poorly controlled ascites; patients with a history of liver transplantation; patients with a history or complication of other liver disease such as hepatitis B or C virus infection, primary sclerosing cholangitis, alcoholic liver injury, autoimmune hepatitis or metabolic dysfunction associated steatohepatitis (MASH). Other patients who were considered ineligible by the investigator or subinvestigator were excluded.

### 2.3 Treatment procedures

This study consisted of a Screening Period, Randomized Period (12-week Double-Blind Phase and 52-week Open-Label Phase) and 70-day Follow-up Period (Supporting Fig. 1). The Double-Blind Phase consisted of Part A, which aimed to confirm the proof of concept, and Part B, which aimed to confirm the dose response. E6011 was administered as an intravenous infusion for about 30 min. In Part A, subjects were randomly assigned to the E6011 10 mg/kg/eow group or placebo group (20 patients at 3:1 ratio). Interim analysis was performed by the independent data monitoring committee (IDMC; IDMC has three members. It's secretariat commissioned to EPS Corporation, Tokyo, Japan) in unblinded conditions when the efficacy and safety data for Week 12 have been obtained for subjects enrolled in Part A. After the completion of the enrollment for Part A, the study proceeded to Part B. In Part B, the subjects were randomly assigned to the placebo group, 10 mg/kg/month group, 15 mg/kg/month group, or the E6011 10 mg/kg/eow group (60 patients), totally falling into 80 patients at 1:1:1:1 ratio (20 patients per each group in the sum of Part A and Part B). In the Open-Label Phase, the subjects were assigned to either the E6011 15 mg/kg/month group or the 10 mg/kg/month at a ratio of 1:1.

The subjects were assigned to dose groups according to the dynamic allocation algorithm prepared by an independent subject enrollment center (Bi-medical Co., Ltd., Tokyo, Japan). Bi-medical Co., Ltd. stored the sealed allocation number table under strict control until unblinding. In the Double-Blind Phase, the ALP value at screening (<3 or ≥3 times the ULN) and the daily dose of UDCA (600 mg or >600 mg) were used as factors for allocation. In the Open-Label Phase, the ALP value at Week 10 (percent change relative to the baseline, <15 % or ≥15 %) was used as a factor for allocation.

The type of prohibited prior and/or concomitant drugs/therapies were as follows (Supporting Fig. 2): the drugs (e.g. bezafibrate) that were suggested to have efficacy on PBC, which were discontinued 12 weeks prior to study treatment; the drugs with evident hepatotoxicity, or live vaccine; biological products, immunoglobulin preparations or blood preparations; other investigational drugs; the drugs with indications for the improvement of hepatic function, cholagogues, interferon preparations, or immunosuppressants; liver transplantation. The use of UDCA was allowed concurrently during the trial, but the changes in dosage and administration were prohibited. About treatment drugs for the underlying disease (pruritus, fatigue, symptoms of dryness, etc.), the type, dosage and administration remained unchanged to the extent possible throughout the study period.

### 2.4 Efficacy assessments

The treatment response as reflected in ALP was confirmed to be associated with survival rate and histological changes of the liver in patients with PBC [[26], [27], [28], [29]]. and it was therefore used widely and globally for the prediction of prognosis in the disorder.

The primary efficacy endpoint was percent change in ALP from the baseline to Week 12 (last observation carried forward [LOCF]). Secondary efficacy endpoints were evaluated using ALP, AST, ALT, gamma-glutamyl transpeptidase (GGT), total bilirubin and total bile acid, Child-Pugh scores [30], the Japanese version of PBC-40 (Symptoms, Itch, Fatigue, Cognitive, Emotional, and Social) [31], hepatic histological evaluation based on the Nakanuma classification [32] (scores of fibrosis, disappearance of bile duct and orcein-positive granule deposits, disease stage based on the total score, cholangitis activity and hepatitis activity; Supporting Table 1), and Fibrosis-4 (Fib-4) index [33].

Laboratory tests were performed by LSI Medience Corporation (Tokyo, Japan) and Cosmic Corporation Co., Ltd. (Tokyo, Japan). Hepatic histological staining (liver biopsy) was performed by Sapporo General Pathology Laboratory Co., Ltd. and GeneticLab Co., Ltd. Hepatic histological evaluation was done centrally by central histopathological committee (three experts in pathology; KH, YK, YN).

### 2.5 Pharmacokinetic and pharmacodynamics assessments

Serum E6011 concentration was measured as PK evaluation. Serum total FKN concentration and CD16-positive monocytes such as blood CX3C motif chemokine receptor 1 (CX3CR1)-positive cells were assessed as biomarkers. CX3CR1 was highly expressed on CD16 positive monocytes, and the decrease of the number of CD16 positive monocytes has the potential as a PD marker of E6011. Serum anti-E6011 antibody (ADA) was investigated as immunogenicity evaluation. Serum E6011 concentrations and serum ADAs were determined by LSI Medience Corporation (Tokyo, Japan). Serum concentrations of total FKN were determined by SEKISUI MEDICAL Co., Ltd (Ibaraki, Japan). Blood CX3CR1-positive cells were measured by KAN Research Institute, Inc. (Kobe, Japan).

### 2.6 Safety assessments

Adverse events (AEs) were gathered based on clinical laboratory tests, vital signs, chest x-ray, standard 12-lead electrocardiograms (ECGs), and neurological findings including progressive multifocal leukoencephalopathy (PML) [[34], [35], [36]]. The investigator or subinvestigator took all measures to classify each AE according to its severity and the causal relationship with the investigational drug. The AEs occurring until 70 days after the last dose were included.

### 2.7 Statistical analysis

In this study, one interim analysis was performed by the IDMC for the purpose of efficacy and safety evaluations. With respect to efficacy, the posterior distribution of the difference (δ) between the treatment groups (an E6011 10 mg/kg/eow group – the placebo group) in percent change in ALP from the baseline to Week 12 (primary endpoint) was calculated using a Bayesian approach. If the posterior probability that percent change in ALP in the E6011 group was a decrease of less than 10 % compared to the placebo group (δ > −10 %) was 90 % or higher at the time of the interim analyses, this treatment was judged to be ineffective, and the study was discontinued.

As this study was discontinued due to a lack of the efficacy according to one interim analysis, the efficacy analysis was performed in the following. Efficacy analyses were performed using the full analysis set (FAS), defined as all subjects who received at least one dose of the study drug and had evaluable data for the primary efficacy endpoint at 1 or more time points. The percent change from baseline to Week 12 in ALP was summarized using summary statistics (mean, standard deviation, median, and range). The difference from the placebo group and its 95 % confidence interval (CI) were also calculated. E6011 10 mg/kg/eow group and the placebo group were compared using one-sided *t*-test. Each E6011 group and the placebo group were compared using a *t*-test and a one-sided significance level (α) of 0.025 with respect to secondary efficacy endpoints. The difference from the placebo group and its 95 % CI were also calculated. For each of these, the measurements and the amount of change and percent change from baseline were summarized using summary statistics (mean, standard deviation, median, and range). Other analyses were performed using Safety Analysis Set. The Safety Analysis Set was the group of subjects who received the investigational drug at least 1 and received a safety evaluation after administration of the investigational drug at least once. The AE verbatim descriptions (investigator terms from the CRF) were classified into standardized medical terminology using the Medical Dictionary for Regulatory Activities (MedDRA).

## 3 Results

### 3.1 Subject dispotion, demographics and baseline characteristics

**Fig. 1:**
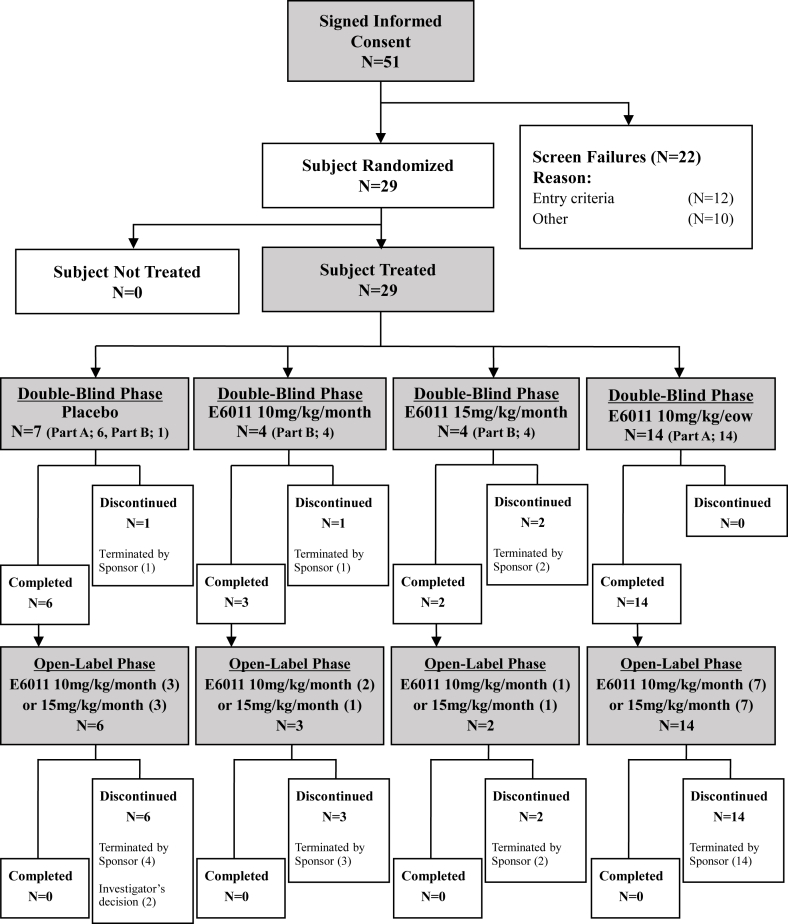
Subject Disposition and Primary Reason for Discontinuation From Study 51 subjects were screened, 29 subjects received the study drug of the 12-week Double-Blind Phase (20 in Part A, 9 in Part B) and 25 subjects entered the 52-week Open-Label Phase. eow = every other week.

Fifty-one subjects were screened, 29 received the study drug of the 12-week Double-Blind Phase (20 in Part A, 9 in Part B) and 25 subjects entered the 52-week Open-Label Phase (Fig. 1). Except for 2 subjects during the Open-Label Phase who were discontinued by the investigator's decision (because of inadequate therapeutic effect and progression of disease respectively), remaining 27 subjects, namely 4 during the Double-Blind Phase and 23 during the Open-Label Phase, were discontinued according to the results of interim analysis (data not shown). The sponsor terminated the study in the middle of Part B due to the lack of the efficacy based on IDMC's recommendations. All of the randomized subjects (29 subjects) were included in the FAS or Safety Analysis Set. The Per Protocol Set included 28 subjects. One subject in the placebo group was excluded due to complication of MASH.

Demographics and baseline characteristics is presented in Table 1. Mean age was 55 years, the majority of subjects were female (90 %) and the mean PBC duration was 6.5 years. Approximately 66 % of subjects received a dose of UDCA of 13 mg/kg/day or higher. Mean ALP was 1003 U/L (normal range, 100–325 U/L) and approximately 41 % subjects of screening visit had ALP more than or equal to 3 × ULN. The mean AST and ALT were 60 U/L (normal range, 10–40 U/L) and 57 U/L (normal range, 5–45 U/L), respectively. Anti-mitochondrial M2 antibodies were positive in 90 % of subjects.

**Table 1:** Demographics and baseline characteristics (FAS).

Category	Placebo (N = 7)	**E6011**	**E6011**	**E6011**	**Overall** (N = 29)
		**10 mg/kg/month** (N = 4)	**15 mg/kg/month** (N = 4)	**10 mg/kg/eow** (N = 14)	
Age (yr), Mean ± SD	56 ± 13	58 ± 15	45 ± 6	56 ± 11	55 ± 12
Female sex, n (%)	6 (86)	4 (100)	4 (100)	12 (86)	26 (90)
Weight (kg), Mean ± SD	57 ± 8	53 ± 8	57 ± 7	58 ± 7	57 ± 7
PBC duration (yr),	8.2 ± 9.4	12.8 ± 15.1	3.1 ± 2.2	4.9 ± 4.3	6.5 ± 7.9
Mean ± SD					
Dose of UDCA n (%)					
600 mg/day	3 (43)	1 (25)	1 (25)	5 (36)	10 (34)
>600 mg/day	4 (57)	3 (75)	3 (75)	9 (64)	19 (66)
<13 mg/kg/day	3 (43)	0 (0)	1 (25)	6 (43)	10 (34)
13–15 mg/kg/day	3 (43)	1 (25)	2 (50)	2 (14)	8 (28)
>15 mg/kg/day	1 (14)	3 (75)	1 (25)	6 (43)	11 (38)
ALP (U/L), Mean ± SD	987 ± 259	794 ± 180	976 ± 275	1080 ± 461	1003 ± 366
ALP at screening, n (%)					
<3 x ULN	4 (57)	3 (75)	2 (50)	8 (57)	17 (59)
≥3 x ULN	3 (43)	1 (25)	2 (50)	6 (43)	12 (41)
T-Bil (mg/dL), Mean ± SD	1.2 ± 0.4	0.9 ± 0.5	1.0 ± 0.3	0.9 ± 0.4	1.0 ± 0.4
AST (U/L), Mean ± SD	64 ± 22	46 ± 13	61 ± 11	62 ± 29	60 ± 24
ALT (U/L), Mean ± SD	71 ± 56	43 ± 16	60 ± 21	54 ± 39	57 ± 39
GGT (U/L), Mean ± SD	375 ± 318	212 ± 149	720 ± 662	324 ± 222	375 ± 341
Total Bile Acid (μmol/L),	91 ± 169	28 ± 10	39 ± 24	44 ± 45	52 ± 87
Mean ± SD					
Fib-4 index, Mean ± SD	3.73 ± 2.56	3.58 ± 2.58	2.59 ± 1.90	2.85 ± 1.85 (N = 12)	3.15 ± 2.09 (N = 27)
AMA, positive n (%)	6 (86)	2 (50)	4 (100)	10 (71)	22 (76)
AMA-M2, positive n (%)	7 (100)	3 (75)	4 (100)	12 (86)	26 (90)
Anti-centromere antibodies, positive n (%)	1 (14)	2 (50)	0 (0)	3 (21)	6 (21)
Anti-GP210 antibodies, positive n (%)	4 (57)	2 (50)	3 (75)	9 (64)	18 (62)
Child-Pugh score	5.4 ± 0.8	5.3 ± 0.5	5.0 ± 0.0	5.0 ± 0.0	5.1 ± 0.4
PBC40, Total Mean ± SD	74 ± 18	100 ± 4	76 ± 14	80 ± 21	81 ± 19
PBC40, Itch Mean ± SD	6.6 ± 4.8	9.0 ± 1.8	8.8 ± 3.5	5.6 ± 3.4	6.8 ± 3.7

ULN = upper limit of normal.

Normal range; ALP (100–325 U/L), T-Bil (0.2–1.2 mg/dL), AST (10–40 U/L), ALT (5–45 U/L), GGT (≤30 U/L), Total Bile Acid (≤10.0 μmol/L), Fib-4 index is calculated as [age (year) × AST (U/L)]/[platelet ( × 10^4^/μL) × √ALT (U/L)].

### 3.2 Liver histology

Liver histology according to the Nakanuma classification (Supporting Table 1) at baseline is presented in Table 2. Most randomized subjects (83 %) were Stage 4 (advanced progression) in disease stage. 17 % were Score 3 of fibrosis (liver cirrhosis), 72 % were Score 3 of bile duct loss (bile duct loss in >2/3 of portal tracts) and 72 % were Score 3 of deposition of Orcein-positive granules. 52 % were CA0 (no activity) in cholangitis activity. These data suggested that intrahepatic cholangitis already burned out, resulting in bile duct loss in most of the subjects at baseline. Hepatic histological evaluations at the end were not performed because this study was interrupted according to the interim analysis.

**Table 2:** Liver Histology Characteristics according to the Nakanuma classification at baseline (FAS).

Category	Placebo (N = 7)	**E6011** **10 mg/kg/month** (N = 4)	**E6011** **15 mg/kg/month** (N = 4)	**E6011** **10 mg/kg/eow** (N = 14)	**Overall** (N = 29)
Disease stage, n (%)					
Stage 1	0	0	0	0	0
Stage 2	0	0	0	1 (7)	1 (3)
Stage 3	1 (14)	1 (25)	0	2 (14)	4 (14)
Stage 4	6 (86)	3 (75)	4 (100)	11 (79)	24 (83)
**Fibrosis score**					
Score 0	0	0	0	0	0
Score 1	4 (57)	2 (50)	2 (50)	4 (29)	12 (41)
Score 2	1 (14)	2 (50)	1 (25)	8 (57)	12 (41)
Score 3	2 (29)	0	1 (25)	2 (14)	5 (17)
**Bile duct disappearance score**					
Score 0	0	0	0	1 (7)	1 (3)
Score 1	0	0	0	1 (7)	1 (3)
Score 2	2 (29)	2 (50)	0	2 (14)	6 (21)
Score 3	5 (71)	2 (50)	4 (100)	10 (71)	21 (72)
**Orcein-positive granule deposit score**					
Score 0	0	0	0	0	0
Score 1	0	0	0	1 (7)	1 (3)
Score 2	0	2 (50)	1 (25)	4 (29)	7 (24)
Score 3	7 (100)	2 (50)	3 (75)	9 (64)	21 (72)
**Cholangitis activity, n (%)**					
CA0	3 (43)	2 (50)	3 (75)	7 (50)	15 (52)
CA1	2 (29)	1 (25)	0	2 (14)	5 (17)
CA2	1 (14)	0	0	0	1 (3)
CA3	1 (14)	1 (25)	1 (25)	5 (36)	8 (28)
**Hepatitis activity, n (%)**					
HA0	0	0	0	0	0
HA1	1 (14)	2 (50)	1 (25)	3 (21)	7 (24)
HA2	5 (71)	2 (50)	2 (50)	7 (50)	16 (55)
HA3	1 (14.3)	0	1 (25)	4 (29)	6 (21)

### 3.3 Efficacy

**Fig. 2:**
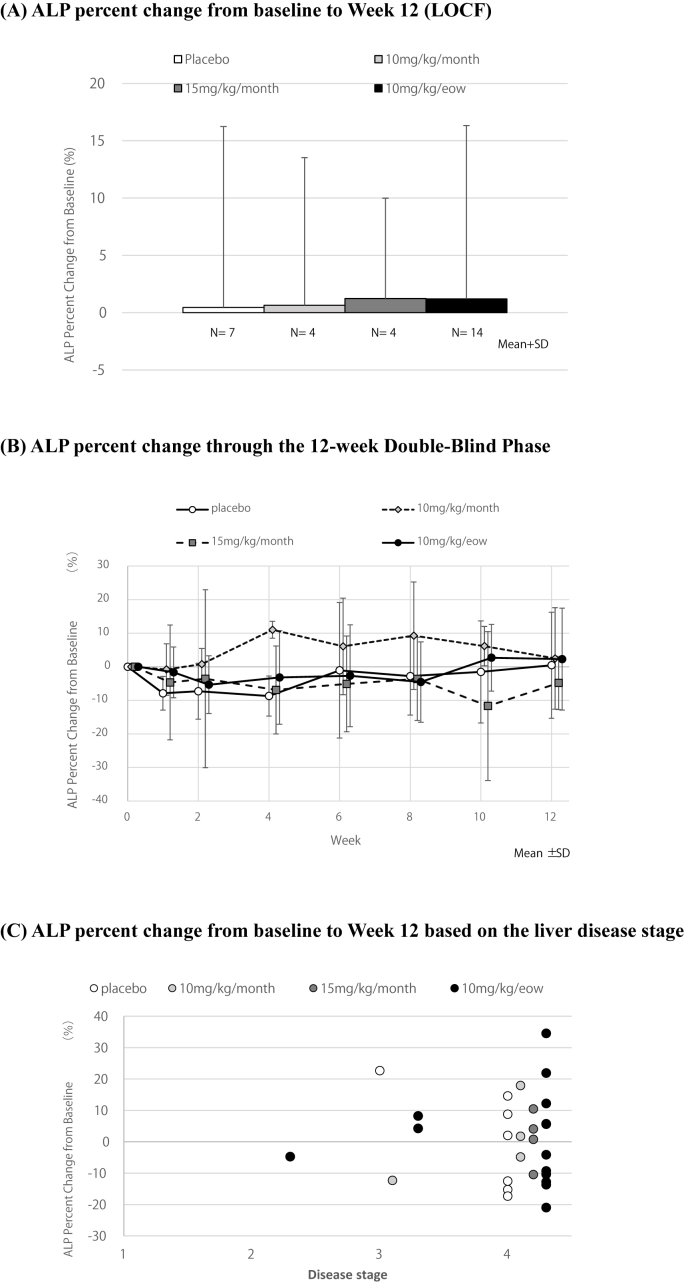
ALP percent change in the 12-week Double-Blind Phase (A) ALP percent change (mean + SD, column chart) from baseline to Week 12 (LOCF), (B) ALP percent change (mean ± SD, line graph) through the 12-week Double-Blind Phase, (C) ALP percent change (dot diagram) from baseline to Week 12 based on the liver disease stage.

The primary endpoint, the mean (SD) ALP percent change from baseline to Week 12 (LOCF), was 0.45 % (15.8) in the placebo group, 0.65 % (12.9) in 10 mg/kg/month, 1.23 % (8.8) in 15 mg/kg/month and 1.19 % (15.1) in the E6011 10 mg/kg/eow group (Fig. 2A) and no statistical differences were found between the placebo group and the E6011 10 mg/kg/eow group. Fig. 2B shows the mean (SD) ALP percent change through the 12-week Double-Blind Phase and any particular trends were not found in E6011 treatment groups. The dot diagram of ALP at Week 12 based on the liver disease stage of the Nakanuma classification was also evaluated in Fig. 2C. No efficacy of E6011 treatment groups were observed regardless of liver disease stage. Subgroup analysis (the ALP value at screening; <3 × ULN, ≥3 × ULN) was performed for the primary endpoint and no definitive conclusion was made (data not shown). There were no clear improvement in ALP percent change after the transition to the Open-Label Phase (all active group, either 10 mg/kg/month or 15 mg/kg/month group) (data not shown).

**Fig. 3:**
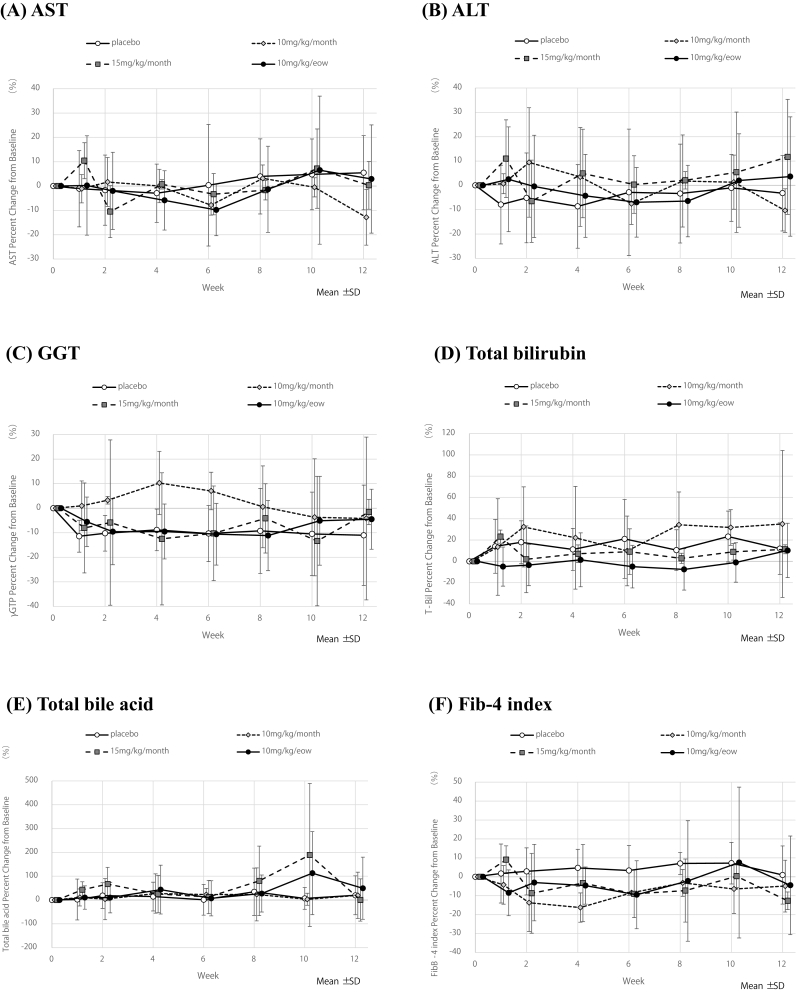
Percent change of the other biochemical parameters through the 12-week Double-Blind Phase Percent change (mean ± SD, line graph) of (A) AST, (B) ALT, (C) GGT, (D) Total bilirubin, (E) Total bile acid and (F) Fib-4 index according to measurement time.

Percent change through the 12-week Double-Blind Phase in AST (Fig. 3A), ALT (Fig. 3B), GGT (Fig. 3C), total bilirubin (Fig. 3D), total bile acid (Fig. 3E), Fib-4 index (Fig. 3F) didn't improve in the E6011 treatment groups and no significant difference were observed from the placebo group. Child-Pugh score didn't change much after 12 weeks in all groups (including the placebo group) from the about 5 points at baseline (data not shown).

The changes from baseline through Week 12 in individual and total score of PBC-40 are summarized in Table 3. No apparent trends related to E6011 administrations were found in PBC-40.

**Table 3:** Change from baseline through week 12 (double-blind phase) in individual and total scores of PBC-40 (FAS).

Category	Placebo (N = 7)	**E6011** **10 mg/kg/month** (N = 4)	**E6011** **15 mg/kg/month** (N = 4)	**E6011** **10 mg/kg/eow** (N = 14)
Total	−0.9 ± 7.0	5.5 ± 6.9	−5.0 ± 10.7	−3.7 ± 10.8
Symptoms	−0.3 ± 1.5	1.5 ± 1.9	−0.3 ± 2.1	−1.1 ± 2.6
Itch	−0.4 ± 2.2	0.0 ± 0.8	−1.3 ± 3.7	0.6 ± 3.0
Fatigue	−1.3 ± 6.4	2.8 ± 3.0	−1.3 ± 2.4	−1.6 ± 5.9
Cognitive	1.4 ± 2.9	1.3 ± 1.0	−2.0 ± 2.2	−1.0 ± 1.5
Emotional	0.0 ± 2.7	0.3 ± 1.3	−1.3 ± 1.7	−0.9 ± 2.3
Social	−0.3 ± 2.5	−0.3 ± 1.9	1.0 ± 5.9	0.3 ± 3.7

Mean ± SD.

### 3.4 Pharmacokinetics and parmacodynamics

**Fig. 4:**
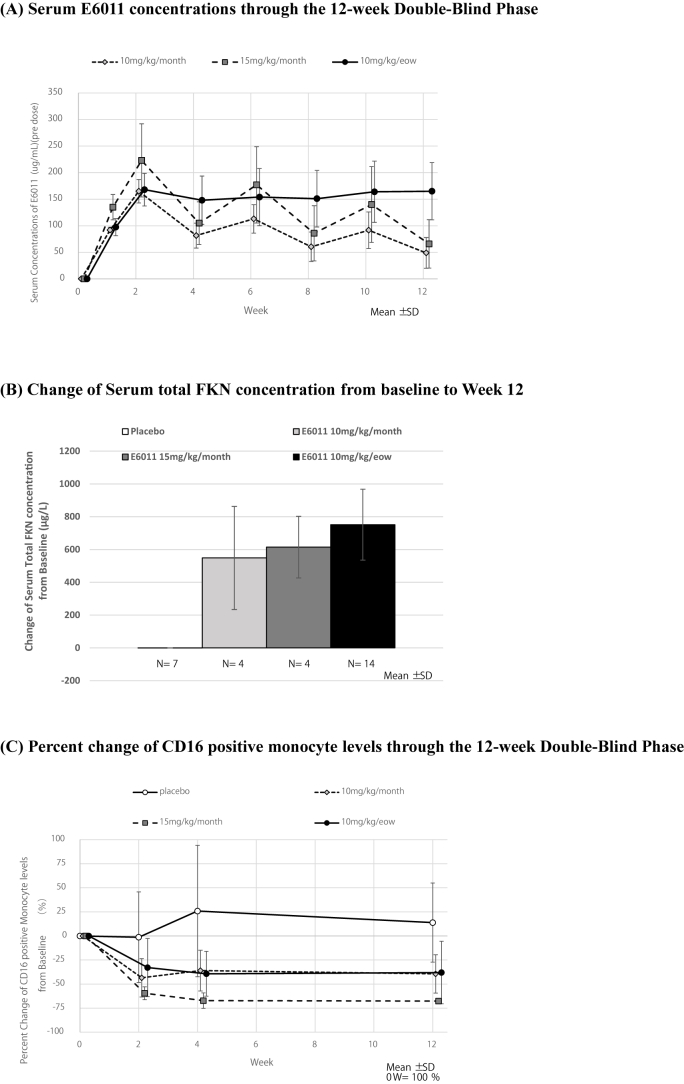
Pharmacokinetics and Pharmacodynamics of E6011 (A) Serum E6011 concentrations (mean ± SD, line graph) through the 12-week Double-Blind Phase, (B) Change of serum total FKN concentration (mean ± SD, column chart) from baseline to Week 12 and (C) Percent change of CD16 positive monocyte levels (mean ± SD, line graph) through the 12-week Double-Blind Phase.

Serum E6011 concentrations appeared to reach a steady state approximately 4–6 weeks after the start of administration at 10 mg/kg/eow. As for the monthly administration groups of 10 mg/kg/month and 15 mg/kg/month, the number of subjects was limited, therefore, it is difficult to judge whether the steady state was reached by Week 12 (Fig. 4A). The relationship between serum E6011 concentrations and ALP percent change was confirmed, but no correlation was found between them (Supporting Fig. 3). Fourteen of 29 subjects (48.3 %) developed anti-E6011 antibodies (ADAs) during the Randomized Period (Double-Blind Phase and Open-Label Phase). We investigated the effect of the neutralizing activity of ADA using chemotaxis assay. In the 10 mg/kg/every-other-week group, which included the highest number of cases (n = 14), we compared E6011 concentration, FKN levels, the percentage of CD16-positive monocytes, and the rate of change in ALP between ADA-positive and ADA-negative groups. The results showed that E6011 concentration and FKN levels were significantly lower in the ADA-positive group, whereas no significant difference was observed in the rate of change in ALP (Supplementary Table 2). The serum total FKN concentration increased after intravenous E6011 administrations compared with baseline (Fig. 4B). This data appeared to reflect the phenomenon that E6011 binds tightly to FKN and FKN was less likely to be metabolized. The FKN receptor, CX3CR1, is highly expressed on CD16 positive monocytes, and the decrease of the number of CD16 positive monocytes is regarded as a PD marker of E6011. CD16 positive monocyte levels (%) in whole monocytes significantly decreased at Week 2 in all of the E6011 treatment groups (Fig. 4C). These results suggested that E6011 definitely inhibited the FKN/CX3CR1 interaction in blood level.

## 4 Safety

Table 4 shows a summary of the adverse events. During the Randomized Period (Double-Blind Phase and Open-Label Phase), a total of 24 subjects (83 %) experienced TEAEs. Of these, 5 subjects (17 %) had treatment-related TEAEs. The most frequently reported TEAE overall was nasopharyngitis (11 subjects, 38 %), followed by contusion (3 subjects, 10 %). Four subjects treated with E6011 had serious TEAEs. Spinal compression fracture occurred in 1 subject in the E6011 10 mg/kg/eow group during the Double-Blind Phase, and esophageal varices haemorrhage (placebo→15 mg/kg/month), hepatic encephalopathy (placebo→10 mg/kg/month), and herpes zoster (10 mg/kg/eow→10 mg/kg/month) occurred in 1 subject each during the Open-Label Phase. Of these, herpes zoster was considered related to the study drug by the investigator and recovered with treatment. No deaths or TEAEs leading to study drug withdrawal occurred in the study. No PMLs were reported during the study.

**Table 4:** Summary of the adverse events during the Randomized Period (Double-Blind Phase and Open-Label Phase) (SAS).

Category	Placebo^#^ (N = 7)	**E6011** **10 mg/kg/month** (N = 4)	**E6011** **15 mg/kg/month** (N = 4)	**E6011** **10 mg/kg/eow** (N = 14)	**Overall** (N = 29)
All TEAEs	7 (100)	2 (50)	3 (75)	12 (86)	24 (83)
≥2 subjects TEAEs					
Constipation	2 (29)	0	0	0	2 (7)
Nasopharyngitis	5 (71)	1 (25)	2 (50)	3 (21)	11 (38)
Upper respiratory tract infection	0	0	0	2 (14)	2 (7)
Contusion	2 (29)	0	0	1 (7)	3 (10)
Treatment-related TEAEs	1 (14)	1 (25)	0	3 (21)	5 (17)
Severe TEAEs	2 (29)	0	0	1 (7)	3 (10)
Serious TEAEs	2 (29)	0	0	2 (14)	4 (14)
Deaths	0	0	0	0	0
TEAEs leading to study drug withdrawal	0	0	0	0	0

n (%), # Subjects received either E6011 10 mg/kg/month or 15 mg/kg/month in the Open-Label Phase.

## 5 Discussion

Although it is a standard policy to treat patients with PBC with UDCA, OCA and PPAR agonists which mainly improve cholestasis, PBC is an autoimmune disease, and a novel therapeutic agent that can suppress the origin of inflammation is needed in order to cure PBC itself fundamentally. E6011 suppresses the leukocyte infiltration into intrahepatic bile ducts in patients with PBC by inhibiting FKN/CX3CR1 axis, and therefore has the potential to manipulate autoimmunity itself. In this hypothesis, we conducted the phase 2a study of the humanized anti-FKN monoclonal antibody E6011 with an incomplete response to UDCA. However, because of a result of the interim analysis, this study was suspended with receiving a recommendation from the IDMC due to lack of efficacy. E6011 failed to decrease ALP levels compared with placebo, and no improvement was observed in the other liver biochemistry values other than ALP.

The causes of failure of the clinical trial were considered from various angles. First of all, inappropriate patients with PBC were enrolled from the viewpoint of liver pathology. Although those with incomplete response to UDCA were enrolled in the study, as other clinical trials in PBC, histopathological findings revealed that most of the participating patients had pathologically advanced disease with bile duct loss, suggesting that the expression of FKN, which is the target of E6011, in the liver tissues might be scarce. Therefore, it is reasoned to assume that the effect of E6011, which suppresses cell migration and accumulation at inflamed tissues, could not be fully exerted in such a patient population. The strong expression of FKN in the bile duct in PBC was reported previously [19], but presumably only in patients with relatively non-progressive disease state (stage1–2). It may be presumed that the efficacy of E6011 could have been exerted if only patients with PBC with high levels of FKN expression in the liver were enrolled. However, many such patients well respond to UDCA, leading to a decrease in ALP and a favourable long-term prognosis. Given the current availability of UDCA as a highly effective, safe, and cost-efficient first-line treatment in PBC, we believe that the decision to target cases with an incomplete response to UDCA in this study was inevitable in terms of feasibility.

Second, the degree of serum E6011 exposure of the liver should be considered. The blood E6011 concentration in this study was similar to that of the Crohn's disease patients who were administered to 10 mg/kg/eow and had good efficacy in previous open studies [25]. However, it is not evident whether the sufficient drug has reached the liver, which is the lesion site of PBC, and the local drug exposure may not be enough for the treatment of patients with PBC.

Third, the lack of efficacy of E6011 may be due to high degree of redundancy present in the chemokine network. In 2018, the result of phase 2a study to investigate the efficacy and safety of NI-0801 was reported [37]. NI-0801 is an anti-chemokine ligand 10 (CXCL10) antibody, and CXCL10 is a chemokine that is secreted by cholangiocytes and attracts inflammatory lymphocytes into the liver via chemokine receptor CXCR3, which are aberrantly expressed on the surface of activated T lymphocytes. Since NI-0801 was expected to inhibit CXCL10-induced infiltration of inflammatory lymphocytes in the vicinity of bile ducts in PBC, the phase 2a study of NI-0801 was conducted. However, any significant improvement of liver chemistries was observed at the end of the NI-0801 administration treatment. The authors of the study discussed that the possible explanation might be the redundancy of chemokine network. In addition to CXCL10, CXCR3 may be able to be activated by other chemokine ligands CXCL9 and CXCL11. Similarly, while E6011 was able to inhibit FKN/CX3CR1 signalling in the current study, CX3CR1 might be activated alternative chemokine apart from FKN.

Finally, it should be discussed whether the primary endpoint in this study was appropriate to evaluate E6011, a compound which exert its effect through inhibition of chemokine network, not with improvement of cholestasis. The majority of approved or investigational drugs for PBC are agents designed to ameliorate cholestasis, making the assessment of their efficacy through a reduction in ALP levels a rational approach. However, in clinical trials of drugs such as E6011, NI-8011, and other biologics that exert therapeutic effects through immunological mechanisms rather than improving cholestasis, it raises the question of whether utilizing ALP as the primary endpoint truly aligns with the nature of these agents. Indeed, in the clinical trials of other biologics, such as ustekinumab (an inhibitor of the IL-12/23 pathway) [38] and abatacept (an inhibitor of the T cell co-stimulatory molecule; CTLA-4) [39], where the anticipated efficacy led to the implementation of clinical trials, a reduction in ALP was set as an endpoint, and these trials were subsequently deemed ineffective. In the context of clinical trials of these biologics, establishment of other endpoints that precisely evaluate their therapeutic effects as well as accurately reflect long-term prognosis is strongly warranted.

Regarding the presence of ADA, although presence of ADA affected E6011 concentration and FKN levels, presumably because ADA was associated with degradation of E6011 and FKN, no significant difference was observed between ADA-positive and -negative groups in the rate of change in ALP (Suppelementary Table 2). Consequently, we concluded that no ADA-positive blood samples showed neutralizing activities and the existence of ADAs did not result in diminished effect of E6011. In terms of safety, we did not observe any significant issue associated with E6011. In fact, we could not fully evaluate the safety of E6011 from the viewpoint of the small number of short administration period, but there were no signs of the increased infectious diseases caused by pharmacological action of E6011.

Several limitations of this clinical trial are noted. The patient with PBC population without bile ducts included in this study may not have been suitable for treatment of E6011. Again, different results may have been achieved in patients with relatively mild PBC who had bile ducts and had high FKN expression. Furthermore, short dosing periods may be the reason why the efficacy of the drug could not be confirmed. Long-term dosing (for example, at least 24 weeks) may be necessary for greater efficacy.

In conclusion, the efficacy of E6011 was not demonstrated in patients with PBC with an incomplete response to UDCA, leading to early termination. Large number of the enrolled patients have shown advanced histological severity at the baseline, which might have contributed to the study results. The future direction of this research will be required to pay attention to both adequate selection of participating patients and trial design suitable for characteristic of E6011.

## CRediT authorship contribution statement

**Atsushi Tanaka:** Writing – review & editing, Investigation, Data curation. **Masanori Abe:** Data curation. **Tadashi Namisaki:** Data curation. **Shinji Shimoda:** Data curation, Conceptualization. **Mikio Zeniya:** Data curation. **Akio Ido:** Data curation. **Hitoshi Yoshiji:** Data curation. **Hiromasa Ohira:** Data curation. **Kenichi Harada:** Investigation, Conceptualization. **Yuko Kakuda:** Investigation, Conceptualization. **Atsushi Umeda:** Investigation, Data curation, Conceptualization. **Yuki Kamiya:** Investigation, Data curation, Conceptualization. **Yukari Higashine:** Investigation, Data curation, Conceptualization. **Seiichiro Hojo:** Supervision. **Toshio Imai:** Supervision, Conceptualization. **Tetsu Kawano:** Supervision, Conceptualization. **Yasuni Nakanuma:** Methodology, Investigation. **Hirohito Tsubouchi:** Supervision, Conceptualization.

## Declaration of competing interest

The authors declare the following financial interests/personal relationships which may be considered as potential competing interests: Atsushi Tanaka reports financial support was provided by EA Pharma Co Ltd. Atsushi Tanaka reports financial support was provided by GSK. Atsushi Tanaka reports financial support was provided by Kowa Company Ltd. Atsushi Tanaka reports financial support was provided by 10.13039/100018736Kaken Pharmaceutical Co Ltd. Atsushi Umeda reports financial support was provided by Ajinomoto Co Inc. Yuki Kamiya reports financial support was provided by Eisai Co Ltd. Yukari Higashine reports financial support was provided by Eisai Inc. Seiichiro Hojo reports financial support was provided by Eisai Inc. Toshio Imai reports financial support was provided by Kan Research Institute Inc. Tetsu Kawano reports was provided by Kan Research Institute Inc. If there are other authors, they declare that they have no known competing financial interests or personal relationships that could have appeared to influence the work reported in this paper.

## Data Availability

Data will be made available on request.

## Appendix

This study was conducted at 46 sites in Japan. The name of principal investigators are as follows: Atsushi Takahashi, Fukushima Medical University Hospital, Fukushima; Atsushi Tanaka, Teikyo University Hospital, Tokyo; Tadashi Namisaki, Nara Medical University Hospital, Nara; Shinji Shimoda, Kyushu University Hospital, Fukuoka; Atsumasa Komori, National Hospital Organization Nagasaki Medical Center, Nagasaki; Akio Ido, Kagoshima University Hospital, Kagoshima; Takeshi Matsui, Teine Keijinkai Hospital, Hokkaido; Yasuhiro Takikawa, Iwate Medical University Hospital, Iwate; Yasushi Matsuzaki, Tokyo Medical University Ibaraki Medical Center, Ibaraki; Satoshi Mochida, Saitama Medical University Hospital, Saitama; Mikio Zeniya, Sanno Medical Center, Tokyo; Akinobu Takaki, Okayama University Hospital, Okayama; Masanori Abe, Ehime University Hospital, Ehime; Shotaro Sakisaka, Fukuoka University Hospital, Fukuoka; Ken Sato, Gunma University Hospital, Gumma; Etsuko Hashimoto and Makiko Taniai, Tokyo Womens Medical University Hospital, Tokyo; Yasunari Nakamoto, University of Fukui Hospital, Fukui; Takeji Umemura, Shinshu University Hospital, Nagano; Masaaki Takamura, Niigata University Medical & Dental Hospital, Niigata; Hiroki Ikeda, Kansai Medical University Hospital, Osaka; Shuhei Hige, Sapporo-Kosei General Hospital, Hokkaido; Yoshiyuki Ueno, Yamagata University Hospital, Yamagata; Satoru Saito, Yokohama City University Hospital, Kanagawa; Ryo Takata, Hyogo College Of Medicine College Hospital, Hyogo; Susumu Tazuma, Hiroshima University Hospital, Hiroshima; Tsutomu Masaki, Kagawa University Hospital, Kagawa; Jun Inoue, Tohoku University Hospital, Miyagi; Kou Nishikawa, Ageo Central General Hospital, Saitama; Yoshiyuki Suzuki, Toranomon Hospital, Tokyo; Shunsuke Nojiri, Nagoya City University Hospital, Aichi; Sawako Uchida, Osaka City University Hospital, Osaka; Takashi Nakahara, Hiroshima University Hospital, Hiroshima; Takumi Kawaguchi, Kurume University Hospital, Fukuoka; Noriko Oza, Saga-Ken Medical Centre Koseikan, Saga; Shigetoshi Fujiyama, Kumamoto Shinto General Hospital, Kumamoto; Hiroshi Sakugawa, Heartlife Hospital, Okinawa; Toshimitsu Murohisa, Dokkyo Medical University Hospital, Tochigi; Kiyotaka Umeki, Chiba-Nishi General Hospital, Chiba; Tatsuji Komatsu, National Hospital Organization Yokohama Medical Center, Kanagawa; Shinji Katsushima, National Hospital Organization Kyoto Medical Center, Kyoto; Tetsuro Takehara, Osaka University Hospital, Osaka; Toshihide Shima, Saiseikai Suita Hospital, Osaka; Tetsuro Inokuma, Kobe City Medical Centar General Hospital, Hyogo; Kenichi Ikejima, Juntendo University Hospital, Tokyo; Jun Itakura, Musashino Red Cross Hospital, Tokyo; Kouichi Takaguchi, Kagawa Prefectural Central Hospital, Kagawa.
