# Supplementary figures and images for: A placebo-controlled Phase 2 trial of E6011, anti-human fractalkine monoclonal antibody, in primary biliary cholangitis
Source: J Transl Autoimmun. 2025 Mar 20;10:100283. doi: 10.1016/j.jtauto.2025.100283 (PMC11986238; doi:10.1016/j.jtauto.2025.100283)

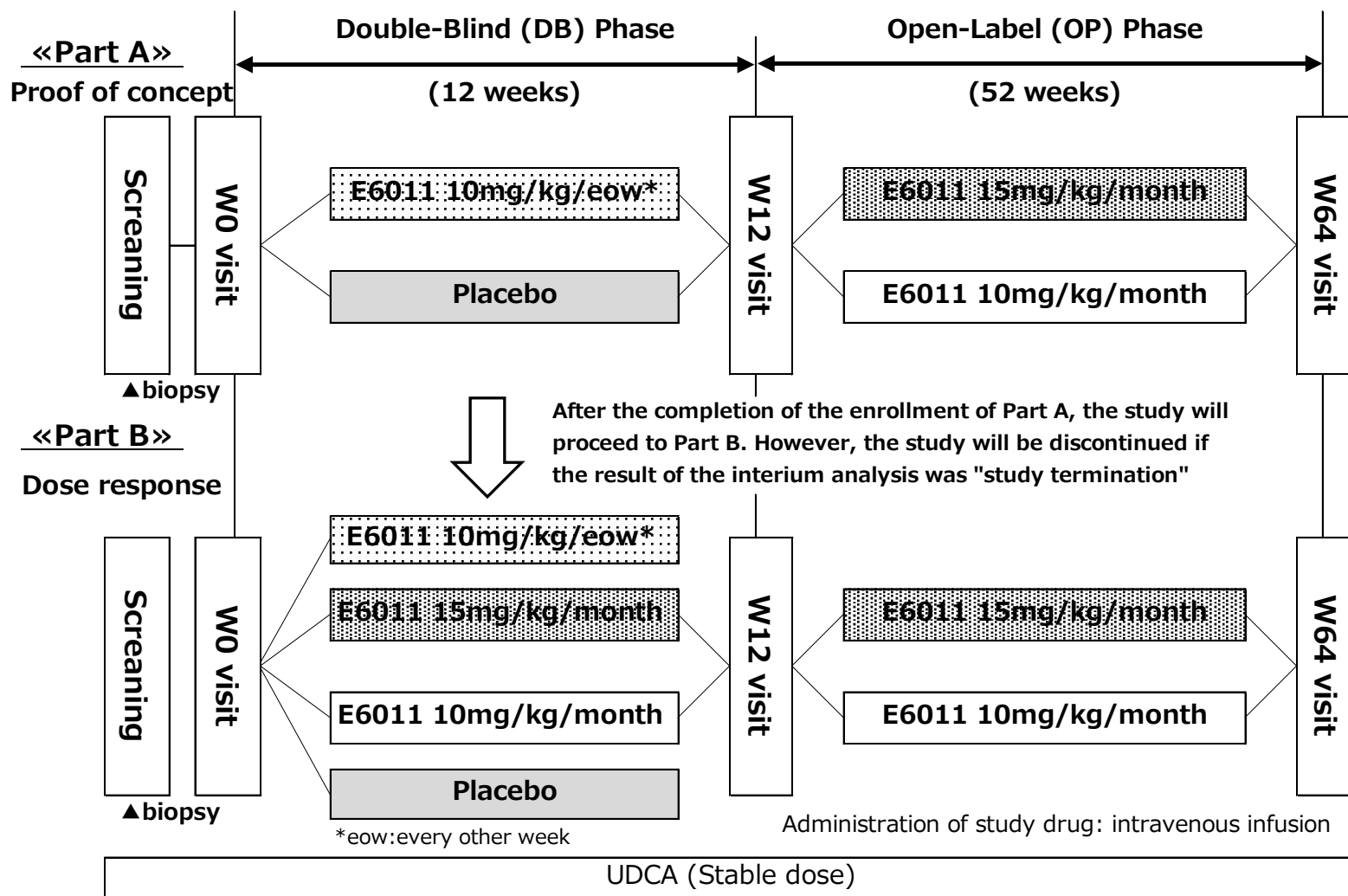

Supplement: Multimedia component 1 [file mmc1.pdf]

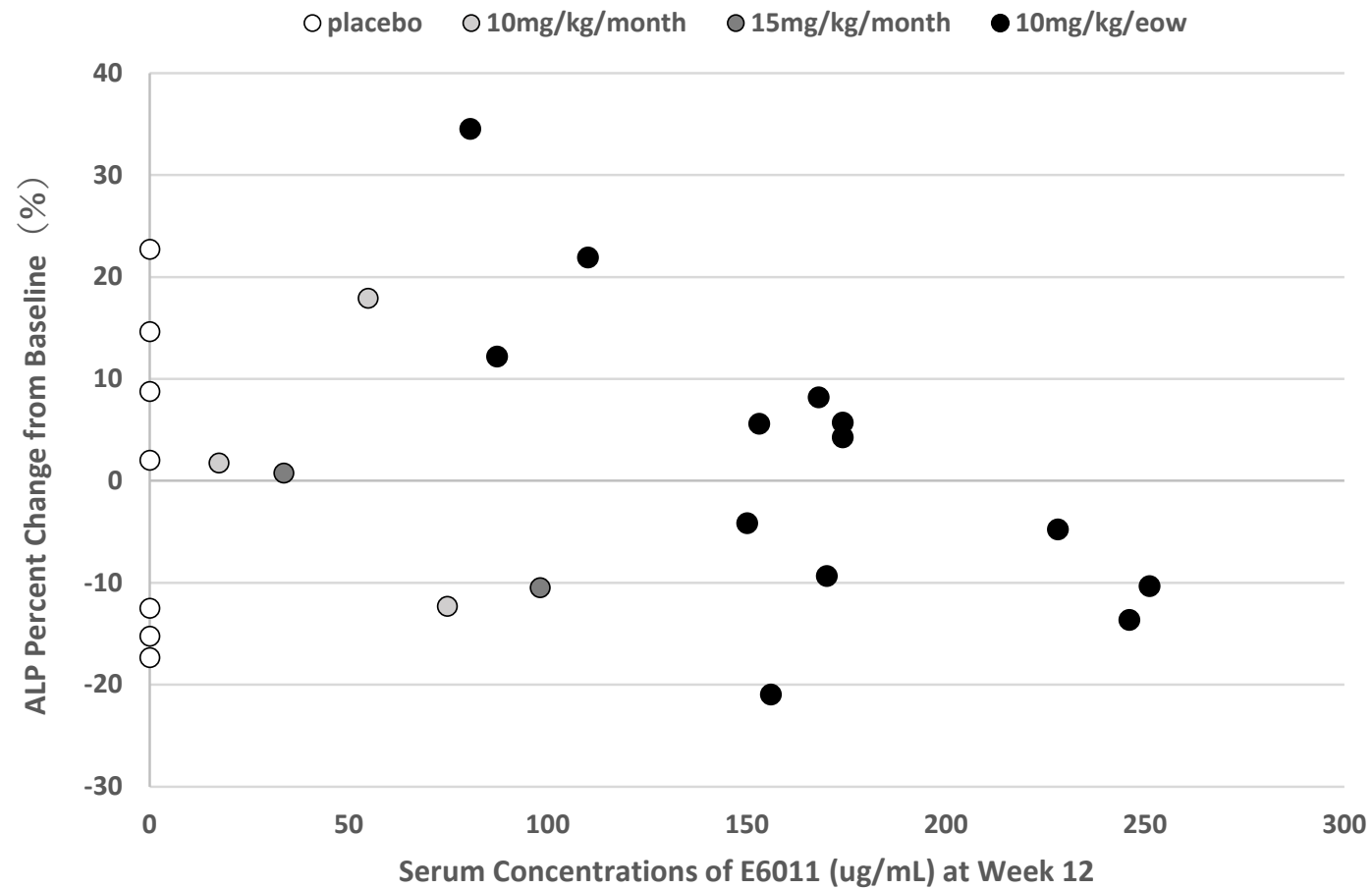

Supplement: Multimedia component 3 [file mmc3.pdf]
