# Supplementary material for: A placebo-controlled Phase 2 trial of E6011, anti-human fractalkine monoclonal antibody, in primary biliary cholangitis
Source: J Transl Autoimmun. 2025 Mar 20;10:100283. doi: 10.1016/j.jtauto.2025.100283 (PMC11986238; doi:10.1016/j.jtauto.2025.100283)
Supplement: Multimedia component 2 [file mmc2.pdf]

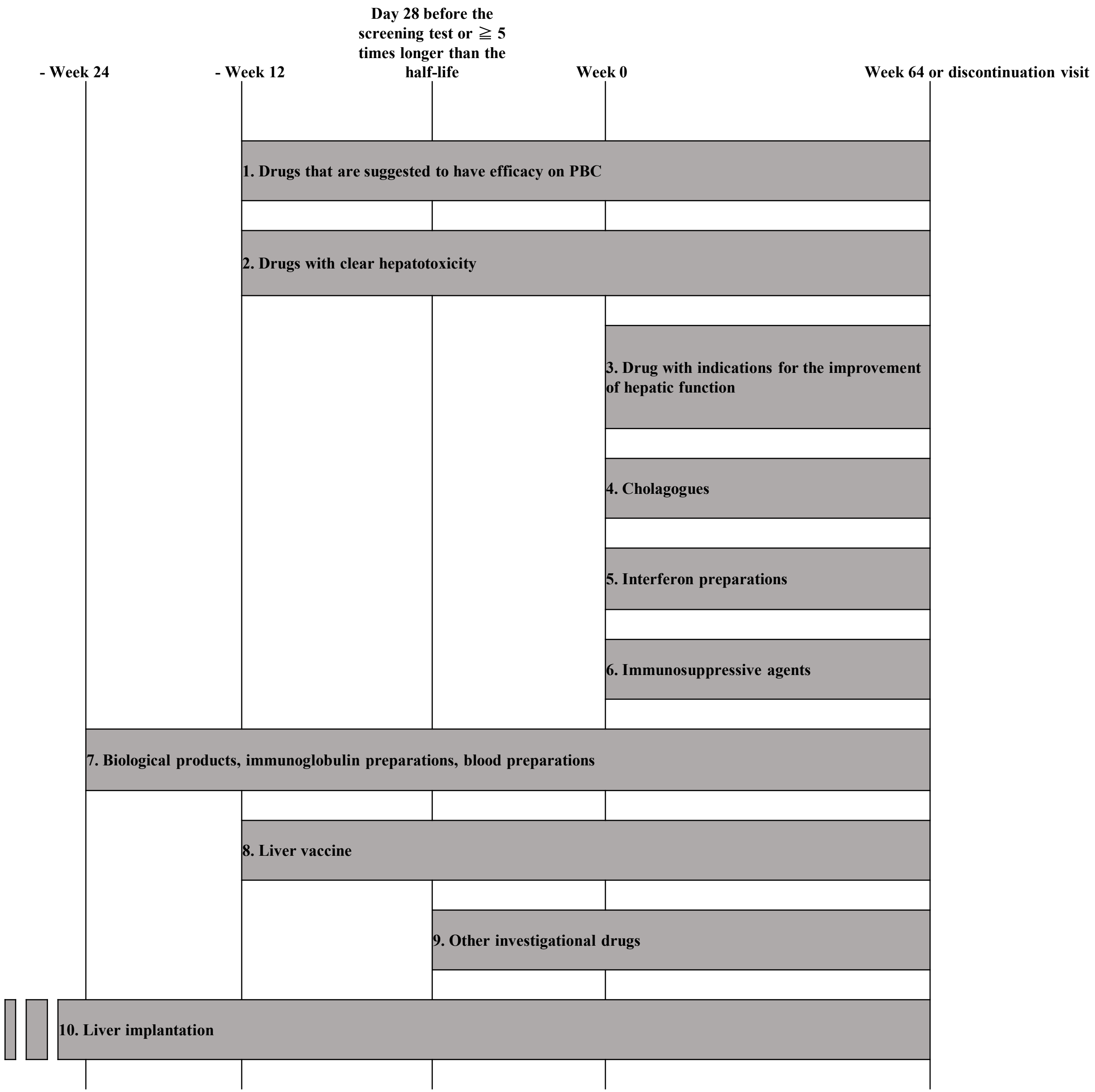
- Week 24
- Week 12
Day 28 before the screening test or ≧ 5 times longer than the half-life
Week 0
Week 64 or discontinuation visit
1. Drugs that are suggested to have efficacy on PBC
2. Drugs with clear hepatotoxicity
3. Drug with indications for the improvement of hepatic function
4. Cholagogues
5. Interferon preparations
6. Immunosuppressive agents
7. Biological products, immunoglobulin preparations, blood preparations
8. Liver vaccine
9. Other investigational drugs
10. Liver implantation
