# Supplementary material for: A placebo-controlled Phase 2 trial of E6011, anti-human fractalkine monoclonal antibody, in primary biliary cholangitis
Source: J Transl Autoimmun. 2025 Mar 20;10:100283. doi: 10.1016/j.jtauto.2025.100283 (PMC11986238; doi:10.1016/j.jtauto.2025.100283)
Supplement: Multimedia component 4 [file mmc4.docx]

**Supporting TABLE. 1. Nakanuma classification (a staging and grading system of the liver for PBC)**

1. Nakanuma Classification (Disease-Stage Classification)

| Scoring of fibrosis | |
| --- | --- |
| Score 0  Score 1  Score 2  Score 3 | No portal fibrosis, or fibrosis limited to portal tracts  Portal fibrosis with periportal fibrosis or incomplete septal fibrosis  Bridging fibrosis with variable lobular disarray  Liver cirrhosis with regenerative nodules and extensive fibrosis |
| Scoring of bile duct loss | |
| Score 0  Score 1  Score 2  Score 3 | No bile duct loss  Bile duct loss in < 1/3 of portal tracts  Bile duct loss in < 1/3 - 2/3 of portal tracts  Bile duct loss in > 2/3 of portal tracts |
| Scoring of deposition of orcein-positive granules | |
| Score 0  Score 1  Score 2  Score 3 | No deposition of granules  Deposition of granules in several periportal hepatocytes in < 1/3 of portal tracts  Deposition of granules in variable periportal hepatocytes in 1/3 - 2/3 of portal tracts  Deposition of granules in many hepatocytes in > 2/3 of portal tracts |
| Sum of score: fibrosis, bile duct loss and deposition of orcein-positive granules | |
| Score 1 (no progression)  Score 2 (mild progression)  Score 3 (moderate progression)  Score 4(advanced progression) | 0  1 - 3  4 - 6  7 - 9 |

1. Nakanuma Classification (Activity Classification)

| Cholangitis activity | |
| --- | --- |
| CA 0 (no activity)  CA1 (mild activity)  CA2 (moderate activity)  CA3 (marked activity) | No cholangitis, but mild duct epithelial damage may be present  One evident chronic cholangitis in the specimen  More than two bile ducts with evident chronic cholangitis  At least one CNSDC in the specimen |
| Hepatitis activity | |
| HA 0 (no activity)  HA1 (mild activity)  HA2 (moderate activity)  HA3 (marked activity) | No interface hepatitis, and no or minimum lobular hepatitis  Interface hepatitis affecting 10 continuous hepatocytes in one portal tract or fibrous septa,  and mild-moderate lobular hepatitis  Interface hepatitis affecting 10 continuous hepatocytes in more than two portal tracts or  fibrous septa, and mild-moderate lobular hepatitis  Interface hepatitis affecting 20 continuous hepatocytes in more than half of the portal  tracts, and moderate lobular hepatitis, or bridging or zonal necrosis |

CA, cholangitis activity; CNSDC, chronic non-suppurative destructive cholangitis; HA, hepatitis activity
