# Supplementary material for: A placebo-controlled Phase 2 trial of E6011, anti-human fractalkine monoclonal antibody, in primary biliary cholangitis
Source: J Transl Autoimmun. 2025 Mar 20;10:100283. doi: 10.1016/j.jtauto.2025.100283 (PMC11986238; doi:10.1016/j.jtauto.2025.100283)
Supplement: Multimedia component 5 [file mmc5.docx]

**Supporting TABLE. 2. Comparison of E6011 concentration, FKN levels, the percentage of CD16-positive monocytes, and the rate of change in ALP at LOCF between ADA-positive and negative patients.**

|  | ADA (+) | ADA (-) | P* |
| --- | --- | --- | --- |
| E6011 concentration | 126.90 ± 43.70 | 182.90 ± 55.72 | <0.05 |
| FKN levels | 630.00 ± 180.87 | 844.25 ± 203.10 | <0.05 |
| CD16-positive monocytes (%) | -53.28 ± 19.08 | -22.73 ± 37.32 | ns |
| Change in ALP | 5.76 ± 18.77 | -2.23 ± 11.92 | ns |

Data are shown as mean ± standard deviation.

ADA, anti-drug antibody; FKN, fractalkine

* Student's t-test was used to compare the ADA-positive and -negative groups.
